# The mortality burden of non-trauma fracture for adults with cerebral palsy

**DOI:** 10.1016/j.bonr.2020.100725

**Published:** 2020-10-07

**Authors:** Daniel G. Whitney, Sarah Bell, Edward A. Hurvitz, Mark D. Peterson, Michelle S. Caird, Karl J. Jepsen

**Affiliations:** a Department of Physical Medicine and Rehabilitation, University of Michigan, 325 E. Eisenhower, Ann Arbor, MI 48108, United States of America; b Institute for Healthcare Policy and Innovation, University of Michigan, 2800 Plymouth Rd., Ann Arbor, MI 48109, United States of America; c Department of Orthopaedic Surgery, University of Michigan, 1540 E Hospital Dr., Ann Arbor, MI 48109, United States of America

**Keywords:** CP, cerebral palsy, NTFx, non-trauma fracture, Cerebral palsy, Non-trauma fracture, Mortality

## Abstract

**Background:**

Individuals with cerebral palsy (CP) manifest skeletal fragility problems early in life, are vulnerable to non-trauma fracture (NTFx), and have a high burden of premature mortality. No studies have examined the contribution of NTFx to mortality among adults with CP. The purpose of this study was to determine if NTFx is a risk factor for mortality among adults with CP and if NTFx exacerbates mortality risk compared to adults without CP.

**Methods:**

Data from 2011 to 2016 Optum Clinformatics® Data Mart and a random 20% sample Medicare fee-for-service were used for this retrospective cohort study. Diagnosis codes were used to identify adults (18+ years) with and without CP, NTFx, and pre-NTFx comorbidities. Crude mortality rates per 100 person years were estimated. Cox regression estimated hazard ratios (HR and 95% confidence interval [CI]) for mortality, comparing: (1) CP and NTFx (CP + NTFx; *n* = 1777); (2) CP without NTFx (CP w/o NTFx; *n* = 12,933); (3) without CP and with NTFx (w/o CP + NTFx; *n* = 433,560); and (4) without CP and without NTFx (w/o CP w/o NTFx; *n* = 6.8 M) after adjusting for demographics and pre-NTFx comorbidities.

**Results:**

The 3-, 6-, and 12-month crude mortality rates were highest among CP + NTFx (12-month mortality rate = 6.80), followed by w/o CP + NTFx (12-month mortality rate = 4.91), CP w/o NTFx (12-month mortality rate = 2.15), and w/o CP w/o NTFx (12-month mortality rate = 0.49). After adjustments, the mortality rate was elevated for CP + NTFx for all time points compared to CP w/o NTFx (e.g., 12-month HR = 1.61; 95%CI = 1.29–2.01), w/o CP + NTFx (e.g., 12-month HR = 1.49; 95%CI = 1.24–1.80), and w/o CP w/o NTFx (e.g., 12-month HR = 5.33; 95%CI = 4.42–6.44). There were site-specific effects (vertebral column, lower extremities) on 12-month mortality.

**Conclusions:**

NTFx is associated with an increase of 12-month mortality risk among adults with CP and compared to adults without CP. Findings suggest that NTFx may be a robust risk factor for mortality among adults with CP.

## 1 Introduction

Cerebral palsy (CP) results from damage to or malformation of the developing brain and is the most common pediatric physical disability (Christensen et al., 2014). Secondary consequences of CP include neuromuscular impairments, suboptimal motor function, and insufficient mechanical loading (Whitney et al., 2017; Johnson et al., 2009). As a result, children with CP manifest skeletal fragility problems, including inadequate development of musculoskeletal mass, architecture, and metabolic health throughout growth, regardless of disability severity (Whitney et al., 2017; Johnson et al., 2009; Modlesky et al., 2015; Whitney et al., 2018a). The skeletal fragility issues early in life likely give rise to problems with skeletal maintenance and preservation throughout the lifespan (Whitney et al., 2018b). Nearly half of adults with CP aged 18 years or older are medically diagnosed with low bone mass (i.e., osteopenia or osteoporosis) (Whitney et al., 2018c), and the risk of fracture is more than double among adults with vs. without CP (Whitney et al., 2019a), even after accounting for osteoporosis and other confounding factors (Whitney et al., 2019b).

Among older adults, skeletal fragility, especially non-trauma fracture (NTFx), is a major contributor to functional disability (Neuman et al., 2014), chronic diseases (Veronese et al., 2017), poor quality of life (Harvey-Kelly et al., 2014), and early mortality (Neuman et al., 2014; Kumar et al., 2018; Uriz-Otano et al., 2016). Moreover, pre-fracture health status is a strong predictor of post-fracture health, function, and survival outcomes (Neuman et al., 2014). As such, adults with CP may have an increased rate of post-NTFx mortality because of their unhealthful aging process (Peterson et al., 2013). Specifically, adults with CP have greater risk of chronic diseases across physiological systems, including cardiometabolic, respiratory, kidney, and liver diseases, as well as a variety of mental health disorders (Whitney et al., 2018b; Whitney et al., 2019c; Whitney et al., 2019d). Adults with CP also have greater risk of premature mortality (Strauss et al., 1999; Ryan et al., 2019). However, the contribution of skeletal fragility to health and survival outcomes for adults with CP is poorly understood.

For children with CP, poor musculoskeletal health (Herskind et al., 2016) typically precedes development of risk factors for chronic disease (Ryan et al., 2014). In adults with CP, the magnitude of the disease burden compared to adults without CP is far greater for skeletal fragility than other chronic diseases (Whitney et al., 2018b; Whitney et al., 2019c). It is possible that early-onset skeletal fragility initiates or exacerbates unhealthful aging (e.g., chronic diseases, premature mortality) for adults with CP through a variety of independent and synergistic mechanisms. A better understanding of the link between skeletal fragility and mortality risk is needed for this vulnerable population. If skeletal fragility does increase risk for mortality among individuals with CP, clinicians caring for patients with CP should be aware of the need for strategies to prevent skeletal fragility and intensively manage NTFx, with the ultimate goal of maximizing healthful aging throughout the lifespan. Accordingly, the objective of this study was to determine if NTFx among adults with CP is associated with greater 12-month mortality rate compared to adults with CP that do not sustain an NTFx and compared to adults without CP that do sustain an NTFx. We hypothesized that adults with CP that sustain an NTFx would have greater post-NTFx 12-month mortality rate compared to both groups, suggesting that: (1) NTFx is a risk factor for 12-month mortality among adults with CP; and (2) NTFx leads to a greater 12-month mortality burden for adults with vs. without CP.

## 2 Methods

### 2.1 Data source

Data from 2011 to 2016 were extracted and combined from administrative claims databases for this retrospective cohort study. Optum Clinformatics® Data Mart Database (OptumInsight™, Eden Prairie, MN, USA) provided information from privately insured beneficiaries that had commercial or Medicare Advantage plans. A random 20% sample of the Medicare fee-for-service database from the Centers for Medicare and Medicaid Services provided information from publicly insured beneficiaries. These claims-based data include all health service utilization (e.g., inpatient, emergency department) for each individual throughout their enrollment. These nationwide databases have been described elsewhere (Whitney et al., 2019e; Whitney, 2019a) including number of beneficiaries covered and eligibility criteria for coverage. Briefly, the Optum Clinformatics® Data Mart Database covers individuals of any income, age, or disability status with commercial or Medicare Advantage health plans. Eligibility requires that the individual either pays for their coverage or the insurance coverage is paid through their employer, and therefore may reflect a slightly more affluent sector of the population. Medicare fee-for-service is a federally administered health insurance and covers adults ≥65 years of age, individuals of all ages with end-stage renal disease, and individuals <65 years of age with certain disabilities, including CP. While combination of both insurance databases covers a wide array of income and disability status indicators, study findings may not be representative of those covered by Medicaid or who are uninsured. Therefore, interpretations should be made within the scope of this sample. Since data are de-identified, the university Institutional Review Board approved this study as non-regulated.

### 2.2 Sample selection

All medical conditions (e.g., CP, fracture, comorbidities) were identified using the International Classification of Diseases, Ninth and Tenth Revision (ICD-9 and ICD-10), Clinical Modification codes to account for the shift in reporting codes on October 1st, 2015. Information regarding how diagnoses were made or by whom (e.g., primary care physician) is not available in administrative claims data.

Adults ≥18 years of age with CP were identified from the Optum and Medicare databases by at least one inpatient, outpatient, or emergency department claim which covered all diagnostic subtypes (e.g., spasticity, quadriplegic, athetoid). Information regarding severity of CP using common clinical measures (e.g., gross motor function classification system) are not available in insurance claims data and more than 70% had “other” or “unspecified” CP (Whitney et al., 2019c), thus not allowing for stratification or statistical adjustment for the clinical subtypes of CP. The comparison group of adults without CP included individuals with no claims for CP from the Optum database. Using Optum enhances the representativeness of our sample of adults without CP, as enrollment criteria for Medicare among individuals under 65 years of age requires permanent disability, such as end-stage renal disease. While no studies have examined the accuracy of identifying adults with CP in administrative claims data, the single claim-based definition has good accuracy for identifying a different pediatric-onset condition using claims data with 99% sensitivity and a positive predictive value of 79% (Reeves et al., 2014).

We defined NTFx as a fracture without trauma codes (e.g., motor vehicle accident) 7 days before to 7 days after the index fracture date, as guided by previous studies (Keshishian et al., 2017; Buchele et al., 2017; Whitney et al., 2019f). Briefly, trauma codes included motor vehicle, air and space transport, water transport, train, and other vehicle accidents, as well as falling down stairs, falls from ladders/scaffolds, and falls from buildings or other structures. Fractures of the vertebral column, hip (including proximal femur), non-proximal femur, tibia/fibula, humerus, ulna/radius, or unspecified location were identified by at least one inpatient, outpatient, or emergency department claim between 2012 and 2015. This time frame was selected to allow for at least one full year of a “look back” period for those that fractured in 2012 to ascertain baseline comorbidity data and at least one full year of follow up for those that fractured in 2015 for the outcome. Using a single claim to capture fracture has excellent accuracy with up to 98% positive predictive value, which is similar or better than other algorithms (e.g., 2+ claims) (Narongroeknawin et al., 2012).

The sample was then categorized based on CP and NTFx status: (1) with CP and NTFx (CP + NTFx); (2) with CP and without NTFx (CP w/o NTFx); (3) without CP and with NTFx (w/o CP + NTFx); and (4) without CP and without NTFx (w/o CP w/o NTFx). The start date of the follow up was the index date of NTFx or a randomly assigned date for those that did not sustain an NTFx. For the latter, we used a uniform distribution (visually inspected by statistician author SB) to randomly assign a date during the individual's enrollment period between January 1, 2012 to December 31, 2015.

We included individuals that had at least 12 full months of continuous enrollment in a health plan prior to their start date of follow up to sequester baseline comorbidity data, as is common practice for administrative claims data analysis (Chang et al., 2012). We excluded individuals if they were < 18 years of age, covered by Health Maintenance Organization plans, or had dual eligibility with Medicaid (Medicare only) because of incomplete Medicare claims. We excluded individuals if they had unknown/missing data for sex or U.S. region (*n* = 71,018, <1% of sample); there was no missing data for age.

### 2.3 Outcome measure

All-cause cumulative mortality from 2012 to 2016 was determined as the number of days from the start date of follow up to date of death, and separated as 3-, 6-, and 12-month time points. From Optum, the date of death was derived from the Date of Death table. This table is kept up-to-date and is sourced by the Death Master File which is maintained by the Social Security Office. For Medicare fee-for-service, the date of death is derived from the Master Beneficiary Summary File or Medicare Provider Analysis and Review file containing the National Death Index information. Given the importance of death information to the function of insurance plans, validity of deaths is expected to be high. For example, >99% of deaths from Medicare have been validated (www.resdac.org).

### 2.4 Covariates

Covariates were selected based on their relevance to adults with CP, fracture, and mortality, as well as availability in the administrative claims databases. Sociodemographic variables included age, sex, race, and U.S. region of residence. Baseline comorbidities were identified from 2011 to 2015 by at least: (1) one inpatient claim within the 12-months prior to the start date of follow up; or (2) two outpatient claims on different claim days within 365 days of one another, with the first outpatient claim occurring within the 12-months prior to the start date of follow up. This claims-based definition generally improves accuracy of identifying “non-event” comorbidities over a single claim (e.g., fracture is an “event”) (Chang et al., 2012). Comorbidities included cardiovascular disease (i.e., ischemic heart disease, heart failure, cerebrovascular disease), hypertension, diabetes (i.e., type 1, type 2), respiratory disease (i.e., acute respiratory infection, pneumonia, chronic obstructive pulmonary disease, interstitial/pleura disease, other respiratory disease), chronic kidney disease (stages I-V), or cancer anywhere in the body, as previously described (Whitney et al., 2019f; Whitney, 2019b).

### 2.5 Statistical analysis

Baseline descriptive characteristics were summarized for each group. Mortality rates (MR) were estimated for each group as the number of deaths divided by the amount of person-years, and expressed per 100 person years. Mortality rate ratios (MRR) and 95% confidence intervals (CI) were estimated comparing each group to one another. Analyses were not performed when the number of deaths for a group was ≤20, as this limits reliability of the point estimate and confidence limits.

Cox regression was used to adjust for covariates when comparing MR, by estimating hazard ratios (HR and 95% CI) of mortality, comparing each group to one another. The primary group comparisons of interest were: (1) CP + NTFx vs. CP w/o NTFx to determine if NTFx is a risk factor for mortality among adults with CP; and (2) CP + NTFx vs. w/o CP + NTFx to determine if NTFx exacerbates MR for adults with vs. without CP. Analyses were not performed when the number of deaths for a group was ≤20. Analyses were performed when the number of deaths for each group was 5–9 per number of covariates. This range provides coverage and bias within acceptable levels similar to the rule of thumb of 10 or more events per number of covariates for Cox models (Vittinghoff and McCulloch, 2007). Covariates were used to explain the difference in crude MRs between groups and were introduced in the following order, depending on the lowest number of deaths among the groups (e.g., if 34 deaths in one group, then the first 5 covariates plus the group variable were used): age, sex, US region, cardiovascular disease, respiratory disease, chronic kidney disease, cancer, diabetes, and hypertension. Possible interactions between exposure status and age or sex were assessed by conducting separate analyses for age or sex strata (to estimate group effects) and by including product terms in the Cox models (to test for interactions). Individuals were right censored if they discontinued health plan enrollment or were alive at the end of the study period. Cox regression did not adjust for race to limit bias due to the extent of missing data from Optum (~14%) and difference in coding race between Optum and Medicare.

We then examined crude MR and MRR and adjusted HR of 12-month mortality by fracture location using the procedures described above to identify site-specific effects. To enhance comparability across sites, we adjusted for the same covariates.

### 2.6 Sensitivity analysis

Due to the observational design, results are subject to bias from unmeasured confounding. In order to estimate the extent of unmeasured confounding for the primary analyses, we computed e-values, which measures the minimum strength of association needed to explain away a specific exposure-outcome association, conditional on the set of covariates (VanderWeele and Ding, 2017). We used the highest level covariate-adjusted Cox regression for each analysis.

Analyses were performed using SAS version 9.4 (SAS Institute, Cary, NC, USA) and *P* ≤ 0.05 (two-tailed) was used to determine statistical significance.

## 3 Results

Baseline descriptive characteristics of CP + NTFx (*n* = 1777), CP w/o NTFx (*n* = 12,933), w/o CP + NTFx (*n* = 433,560), and w/o CP w/o NTFx (*n* = 6,830,332) is presented in Table 1. Notably, the two NTFx groups were of similar age and the two non-NTFx groups were of similar age, but were about 10–14 years younger on average than the two NTFx groups. For the CP groups, 53.4% of the CP + NTFx group and 60.3% of the CP w/o NTFx group were covered by Optum, with the remaining 46.4% and 39.7%, respectively, covered by Medicare fee-for-service.

**Table 1:** Baseline descriptive characteristics by status of cerebral palsy (CP) and non-trauma fracture (NTFx).

	CP + NTFx (*n* = 1777)	CP w/o NTFx (*n* = 12,933)	w/o CP + NTFx (n = 433,560)	w/o CP w/o NTFx (n = 6,830,332)
	% (n)	% (n)	% (n)	% (n)
Demographic characteristics				
Age, mean (SD)	63.6 (16.8)	53.0 (18.9)	65.1 (18.4)	51.7 (18.5)
18–40 years	11.2 (199)	27.8 (3592)	12.5 (54,379)	30.4 (2,074,329)
41–64 years	35.6 (633)	40.3 (5207)	28.4 (123,314)	41.1 (2,805,412)
≥65 years	53.2 (945)	32.0 (4134)	59.0 (255,867)	28.6 (1,950,591)
Sex				
Women	56.7 (1008)	47.0 (6081)	66.1 (286,561)	51.4 (3,511,050)
Men	43.3 (769)	53.0 (6852)	33.9 (146,999)	48.6 (3,319,282)
Race				
White	78.6 (1397)	72.5 (9381)	68.9 (298,538)	63.7 (4,349,627)
Black	6.0 (106)	9.6 (1245)	6.6 (28,609)	8.5 (577,185)
Hispanic	5.5 (97)	6.5 (841)	7.9 (34,295)	9.7 (664,785)
Asian	1.5 (27)	1.8 (233)	2.4 (10,565)	4.2 (286,115)
Unknown/missing	8.4 (150)	9.5 (1233)	14.2 (61,553)	13.9 (952,620)
US region				
West	24.1 (429)	21.5 (2785)	28.1 (121,891)	24.1 (1,644,363)
Midwest	22.4 (398)	25.4 (3281)	24.4 (105,860)	25.2 (1,717,875)
South	37.3 (663)	39.1 (5061)	36.2 (157,128)	40.5 (2,766,468)
Northeast	16.2 (287)	14.0 (1806)	11.2 (48,681)	10.3 (701,626)
Comorbidities				
Cardiovascular disease^a^	27.0 (479)	14.3 (1852)	22.3 (96,514)	7.9 (540,149)
Hypertension	53.3 (947)	36.7 (4747)	52.1 (225,711)	27.7 (1,889,368)
Diabetes	20.9 (371)	14.7 (1905)	20.1 (87,046)	11.5 (788,545)
Respiratory disease^b^	30.1 (534)	21.6 (2790)	27.8 (120,346)	14.9 (1,018,564)
Chronic kidney disease	10.1 (180)	4.8 (623)	10.6 (46,058)	3.5 (241,644)
Cancer	15.9 (282)	11.7 (1508)	19.3 (83,711)	11.3 (772,029)
Fracture distribution^c^				
Unspecified location	2.5 (44)	0	4.5 (19,345)	0
Vertebral column	28.0 (498)	0	25.5 (110,532)	0
Hip	23.5 (418)	0	16.9 (73,059)	0
Femur, non-proximal	6.2 (111)	0	3.1 (13,544)	0
Tibia/fibula	24.6 (437)	0	22.7 (98,523)	0
Humerus	11.6 (207)	0	9.9 (42,736)	0
Ulna/radius	6.1 (108)	0	19.1 (82,631)	0

a Ischemic heart disease, heart failure, and/or cerebrovascular disease.

b Acute respiratory infection, pneumonia, chronic obstructive pulmonary disease, interstitial/pleura disease, and/or other respiratory disease (e.g., respiratory failure).

c Some individuals had an NTFx across multiple sites.

### 3.1 Crude mortality rate

The crude MR for all time points were highest for CP + NTFx (e.g., 12-month MR = 6.80), followed by w/o CP + NTFx (e.g., 12-month MR = 4.91), CP w/o NTFx (e.g., 12-month MR = 2.15), and w/o CP w/o NTFx (e.g., 12-month MR = 0.49) (Table 2). The MR remained relatively stable for the two non-NTFx groups from 3-months to 12-months, but declined in the two NTFx groups.

**Table 2:** Crude mortality rate and rate ratio (RR) by status of cerebral palsy (CP) and non-trauma fracture (NTFx).

	3-month mortality	6-month mortality	12-month mortality
Mortality cases	N	N	N
w/o CP w/o NTFx	7454	14,923	29,823
w/o CP + NTFx	9341	13,214	18,223
CP w/o NTFx	84	147	261
CP + NTFx	53	69	109

Crude mortality rate	N per 100 person years	N per 100 person years	N per 100 person years
w/o CP w/o NTFx	0.45	0.46	0.49
w/o CP + NTFx	9.02	6.64	4.91
CP w/o NTFx	2.64	2.35	2.15
CP + NTFx	12.35	8.24	6.80

Crude mortality RR	RR (95% CI)	RR (95% CI)	RR (95% CI)
Reference: w/o CP w/o NTFx			
w/o CP + NTFx	20.07 (19.47, 20.69)	14.36 (14.02, 14.70)	10.05 (9.86, 10.23)
CP w/o NTFx	5.88 (4.74, 7.29)	5.07 (4.31, 5.97)	4.39 (3.89, 4.96)
CP + NTFx	27.51 (21.00, 36.04)	17.83 (14.07, 22.58)	13.91 (11.53, 16.79)
Reference: w/o CP + NTFx			
CP w/o NTFx	0.29 (0.24, 0.36)	0.35 (0.30, 0.42)	0.44 (0.39, 0.49)
CP + NTFx	1.37 (1.05, 1.80)	1.24 (0.98, 1.57)	1.38 (1.15, 1.67)
Reference: CP w/o NTFx			
CP + NTFx	4.68 (3.32, 6.60)	3.51 (2.64, 4.68)	3.17 (2.53, 3.96)

The crude MRR was statistically elevated for CP + NTFx compared to CP w/o NTFx for all time points, and statistically elevated compared to w/o CP + NTFx for 3-months and 12-months, but not statistically elevated for 6-months; although, the crude MRR for 6-months was marginally insignificant (MRR = 1.24; 95% CI = 0.98–1.57) (Table 2).

### 3.2 Cox regression analysis of mortality

Compared to w/o CP w/o NTFx, the HR adjusting for all covariates was highest for CP + NTFx across all time points (e.g., 12-month HR = 5.33; 95% CI = 4.42–6.44), followed by w/o CP + NTFx (e.g., 12-month HR = 3.60; 95% CI = 3.53–3.67) and CP w/o NTFx (e.g., 12-month HR = 3.31; 95% CI = 2.93–3.74) (Table 3). The adjusted HR comparing CP + NTFx to CP w/o NTFx was elevated for all time points with the group effect becoming smaller from 3-months (HR = 2.37; 95% CI = 1.68–3.34) to 12-months (HR = 1.61; 95% CI = 1.29–2.01). The adjusted HR comparing CP + NTFx to w/o CP + NTFx was elevated for all time points (e.g., 12-month HR = 1.49; 95% CI = 1.24–1.80).

**Table 3:** Adjusted hazard ratio (HR) of mortality by status of cerebral palsy (CP) and non-trauma fracture (NTFx).

	3-month mortality	6-month mortality	12-month mortality
	HR (95% CI)	HR (95% CI)	HR (95% CI)
Reference: w/o CP w/o NTFx			
w/o CP + NTFx	6.92 (6.70, 7.15)	5.04 (4.91, 5.16)	3.60 (3.53, 3.67)
CP w/o NTFx	4.33 (3.49, 5.36)	3.76 (3.20, 4.42)	3.31 (2.93, 3.74)
CP + NTFx	10.23 (7.80, 13.40)	6.69 (5.28, 8.48)	5.33 (4.42, 6.44)
Reference: w/o CP + NTFx			
CP w/o NTFx	0.62 (0.50, 0.77)	0.75 (0.63, 0.88)	0.92 (0.81, 1.04)
CP + NTFx	1.48 (1.13, 1.94)	1.33 (1.05, 1.68)	1.49 (1.24, 1.80)
Reference: CP w/o NTFx			
CP + NTFx	2.37 (1.68, 3.34)	1.78 (1.34, 2.37)	1.61 (1.29, 2.01)

Adjusted for age, sex, US region, cardiovascular disease, respiratory disease, diabetes, chronic kidney disease, cancer, and hypertension.

### 3.3 Site-specific effect of NTFx on 12-month mortality rate

Table 4 shows the MR, MRR, and adjusted HR of 12-month mortality stratified by NTFx location. The site-specific patterns of MR were consistent for the two NTFx groups: hip had the highest MR, followed by vertebral column, upper extremities, and lower extremities. The number of deaths in the CP + NTFx group for upper extremities was insufficient for further analyses (*N* ≤ 20). The crude MRR was elevated for adults with vs. without CP for NTFx of the lower extremities. After adjusting for age, sex, and US region, the HR was elevated for adults with vs. without CP for the vertebral column (HR = 1.39; 95% CI = 1.02–1.89) and lower extremities (HR = 1.95; 95% CI = 1.28–2.97).

**Table 4:** 12-month mortality rate, rate ratio (RR), and adjusted hazard ratio (HR) by status of cerebral palsy (CP) and NTFx location.

	Vertebral column	Hip	Lower extremities	Upper extremities
Mortality cases	N	N	N	N
w/o CP + NTFx	6668	6692	2073	2655
CP + NTFx	40	38	22	13

Crude mortality rate	N per 100 person years	N per 100 person years	N per 100 person years	N per 100 person years
w/o CP + NTFx	7.11	11.32	2.14	2.43
CP + NTFx	9.01	10.33	4.44	4.47

Crude mortality RR	RR (95% CI)	RR (95% CI)	RR (95% CI)	RR (95% CI)
Reference: w/o CP + NTFx				
CP + NTFx	1.27 (0.93, 1.73)	0.91 (0.66, 1.26)	2.07 (1.36, 3.15)	a

Adjusted HR	HR (95% CI)	HR (95% CI)	HR (95% CI)	HR (95% CI)
Reference: w/o CP + NTFx				
CP + NTFx	1.39 (1.02, 1.89)	1.21 (0.88, 1.66)	1.95 (1.28, 2.97)	a

Adjusted for age, sex, and US region. ^a^Sample size insufficient for analysis (N ≤ 20). Some individuals had an NTFx across multiple sites and are represented for each location.

### 3.4 Interactions of group with age and sex

Table 5 shows the MR, MRR, and adjusted HR of mortality by age category (<65 years, ≥65 years) and sex (both *P* for interaction <0.001). CP + NTFx had an elevated MR, MRR, and adjusted HR compared to CP w/o NTFx and w/o CP + NTFx for each age category and sex. The older age category for all groups had a higher absolute MR than the younger age category. The absolute difference in MR for CP + NTFx vs. CP w/o NTFx and w/o CP + NTFx was larger in the older vs. younger age category, but the relative MRR was larger for CP + NTFx vs. CP w/o NTFx and w/o CP + NTFx in the younger vs. older age category. Men had a slightly higher MR for all groups compared to women. The absolute difference in MR and adjusted HR between CP + NTFx and w/o CP + NTFx was similar for men and women. However, the absolute difference in MR and adjusted HR was slightly larger for men than women when comparing CP + NTFx and CP w/o NTFx.

**Table 5:** 12-month mortality rate, rate ratio (RR), and adjusted hazard ratio (HR) by status of cerebral palsy (CP) and non-trauma fracture (NTFx) by age and sex.

	< 65 years	≥ 65 years	Women	Men
Mortality cases	N	N	N	N
w/o CP w/o NTFx	4224	25,599	14,423	15,400
w/o CP + NTFx	1536	16,687	10,788	7435
CP w/o NTFx	78	183	119	142
CP + NTFx	22	87	57	52

Crude mortality rate	N per 100 person years	N per 100 person years	N per 100 person years	N per 100 person years
w/o CP w/o NTFx	0.10	1.42	0.46	0.52
w/o CP + NTFx	1.01	7.62	4.35	6.03
CP w/o NTFx	0.95	4.69	2.08	2.20
CP + NTFx	2.90	10.31	6.19	7.62

Crude mortality RR	RR (95% CI)	RR (95% CI)	RR (95% CI)	RR (95% CI)
Reference: w/o CP w/o NTFx				
w/o CP + NTFx	10.27 (9.68, 10.88)	5.37 (5.27, 5.48)	9.47 (9.23, 9.70)	11.62 (11.30, 11.94)
CP w/o NTFx	9.62 (7.69, 12.03)	3.30 (2.86, 3.82)	4.53 (3.78, 5.42)	4.24 (3.60, 5.01)
CP + NTFx	29.49 (19.40, 44.84)	7.26 (5.88, 8.96)	13.46 (10.38, 17.46)	14.67 (11.17, 19.26)
Reference: w/o CP + NTFx				
CP w/o NTFx	0.94 (0.75, 1.18)	0.61 (0.53, 0.71)	0.48 (0.40, 0.57)	0.37 (0.31, 0.43)
CP + NTFx	2.87 (1.89, 4.38)	1.35 (1.09, 1.67)	1.42 (1.10, 1.85)	1.26 (0.96, 1.66)
Reference: CP w/o NTFx				
CP + NTFx	3.07 (1.91, 4.92)	2.20 (1.70, 2.84)	2.97 (2.17, 4.08)	3.46 (2.52, 4.75)

Adjusted HR	HR (95% CI)	HR (95% CI)	HR (95% CI)	HR (95% CI)
Reference: w/o CP w/o NTFx				
w/o CP + NTFx	10.57 (9.97, 11.21)	4.25 (4.16, 4.34)	3.11 (3.03, 3.19)	4.31 (4.19, 4.43)
CP w/o NTFx	9.53 (7.62, 11.93)	2.67 (2.31, 3.09)	3.30 (2.75, 3.95)	3.33 (2.82, 3.93)
CP + NTFx	29.59 (19.46, 44.99)^a^	5.19 (4.20, 6.40)	4.48 (3.45, 5.81)	6.33 (4.82, 8.31)
Reference: w/o CP + NTFx				
CP w/o NTFx	0.90 (0.72, 1.13)	0.63 (0.54, 0.73)	1.06 (0.89, 1.27)	0.77 (0.65, 0.91)
CP + NTFx	2.80 (1.84, 4.26)^a^	1.22 (0.99, 1.51)	1.44 (1.11, 1.87)	1.47 (1.12, 1.93)
Reference: CP w/o NTFx				
CP + NTFx	3.10 (1.93, 4.98)^a^	1.94 (1.51, 2.51)	1.36 (0.99, 1.86)	1.90 (1.39, 2.62)

Adjusted for age, sex, US region, cardiovascular disease, respiratory disease, diabetes, chronic kidney disease, cancer, and hypertension unless otherwise noted.

a Adjusted for age, sex, and US region.

### 3.5 Sensitivity analysis

The e-value (lower 95% CI) needed to fully explain away the effect for CP + NTFx vs. CP w/o NTFx was 4.17 (2.75) for 3-months, 2.96 (2.01) for 6-months, and 2.60 (1.90) for 12-months. The e-value (lower 95% CI) needed to fully explain away the effect for CP + NTFx vs. w/o CP + NTFX was 2.32 (1.51) for 3-months, 1.99 (1.28) for 6-months, and 2.34 (1.79) for 12-months. Given the large e-values, it appears unlikely that unmeasured confounding largely biased effect estimates.

## 4 Discussion

Findings from this study suggest that adults with CP have greater risk of mortality compared to adults without CP, and NTFx is an additive burden. One main finding of this investigation is that NTFx was associated with mortality within 12 months among adults with CP, suggesting that NTFx is a risk factor for mortality for this population. The other main finding of this investigation was that among those that sustained an NTFx, adults with CP had higher rates of mortality within 12 months compared to adults without CP, suggesting a greater NTFx-attributable mortality burden for this population. Further, while we observed that hip and vertebral NTFx were associated with higher NTFx-related mortality rates compared to extremity sites for adults with and without CP, NTFx of the vertebral column and lower extremities may elicit a greater relative mortality burden for adults with vs. without CP. Taken together, study findings suggest that NTFx, an indicator of skeletal fragility, may be implicated in the pathogenesis of unhealthful aging for adults with CP, with evidence of site-specific effects. For clinicians, more aggressive healthcare monitoring for post-NTFx health complications is needed for patients with CP that sustain an NTFx compared to patients with CP without an NTFx and non-CP patients that sustain an NTFx.

The current findings may be at odds with our previous study that found no direct association between fracture and 1-year mortality for adults with CP (Whitney et al., 2019f). However, there were several limitations in our previous study (Whitney et al., 2019f) that the current study was able to overcome. Our previous study leveraged the private insurance database only and was limited to statistical adjustment for important and potentially confounding variables due to the small sample size, such as diseases to account for health/disease status and to serve as a proxy for severity of CP. We also examined CP only without other neurodevelopmental disabilities, which accounts for a relatively smaller portion of the CP population, may not be representative of the greater population with CP given the high co-occurrence of other developmental conditions, and likely reflects a slightly healthier sector of the CP population given the greater medical complexity in the presence of co-occurring neurodevelopmental disabilities (Reid et al., 2018).

In the current study, the initial crude analysis found that MR was highest among CP + NTFx, followed by w/o CP + NTFx, CP w/o NTFx, and w/o CP w/o NTFx. Interestingly, the non-NTFx groups had a relatively stable MR across the 3-, 6-, and 12-month time points, whereas the two NTFx groups had the highest MR at 3-months and a gradual decline to 12-months. Even with this decline, the MR was greater for all time points compared to their non-NTFx groups. This is consistent with the notion that there may be a unique set of factors associated with short- and long-term mortality post-NTFx (Schousboe, 2017). However, the mechanisms explaining the fracture-induced mortality have yet to be elucidated and may be further confounded by the medical and functional complexities of CP.

We used Cox regression to adjust for covariates to better understand the association of CP, NTFx, and mortality beyond group differences in demographic (e.g., age) and pre-NTFx comorbidity attributes. In our primary comparisons of interest, we found that the adjusted HR of 3-, 6-, and 12-month mortality was higher for CP + NTFx compared to CP w/o NTFx and w/o CP + NTFx. Specifically, adults with CP that sustained an NTFx (CP + NTFx) had a 2.4-fold higher HR of mortality in the 3 months following the NTFx event compared to adults with CP that did not sustain an NTFx (CP w/o NTFx). The adjusted HR declined over the time points, but still remained elevated with a 61% higher adjusted HR of mortality at 12-months. The adjusted HR of mortality for adults with CP that sustained an NTFx compared to adults without CP that sustained an NTFx (w/o CP + NTFx) remained relatively stable, with a 48%, 33%, and 49% higher HR for 3-, 6-, and 12-month time points, respectively.

The NTFx location-stratified analyses may suggest site-specific effects on mortality rate for adults with CP, which is consistent with a previous report (Whitney et al., 2019f). In this study, we found evidence that NTFx of the vertebral column and lower extremities elicits a relative rate of 12-month mortality that is nearly 40% and 2-fold higher, respectively, for adults with vs. without CP. The differential NTFx-mortality association by location may be due to many factors. Individuals with CP exhibit inadequate development of bone and altered biomechanical loading throughout growth (Whitney et al., 2017), and adults with CP have poor skeletal preservation (Whitney et al., 2018b; Whitney et al., 2018c; Whitney et al., 2019b; French et al., 2019a). The chronic burden of skeletal fragility throughout the lifespan may lead to site-specific vulnerability to NTFx (e.g., distal femur is the most common fracture site for children with CP), which may in turn impact survival outcomes or disease trajectories. Better understanding site-specific effects of fracture on health outcomes requires further research, as this may lead to more targeted interventions to mitigate skeletal fragility and fracture risk, as well as the subsequent disease sequela for adults with CP. Although, it is important to note that the mortality rate was still much higher for NTFx of the vertebral column and hip than lower extremities for adults with and without CP. Clinicians treating adults with CP should be aware that NTFx of any location may increase risk of mortality, and aggressive monitoring and treatment interventions may be necessary.

Among older adults, pre-fracture chronic diseases (Katsoulis et al., 2017), functional capacity (Tosteson et al., 2007), and physical capacity (e.g., muscle strength) (Bliuc et al., 2009) are robust risk factors of premature mortality post-fracture, and a follow up fracture further exacerbates mortality rate (Bliuc et al., 2009). The overall physiological stress caused by an NTFx may create an unfavorable biological environment that exacerbates risk for post-NTFx complications and premature mortality, either through direct or indirect mechanisms (Schousboe, 2017). To this end, individuals with CP are at particular risk for post-NTFx complications because of their greater lifetime burden of unhealthful aging. Therefore, adults with CP may already have a more compromised physiological environment and skeletal fragility (e.g., history of NTFx), and sustaining an NTFx may further increase susceptibility to post-NTFx complications.

Moreover, via a variety of mechanisms, NTFx may increase inflammation, vascular calcification, risk of respiratory infection due to a dramatic loss of function (e.g., immobility), and other vascular problems (e.g., venous thromboembolism) that may in turn lead to an increased risk of chronic diseases (Veronese et al., 2017). In the current study, we adjusted for pre-NTFx comorbidities (most are considered chronic diseases) and did not account for post-NTFx comorbidities. While unknown, it is possible that adults with CP are particularly vulnerable to post-NTFx chronic diseases or other acute health complications compared to adults without CP. This may help to explain the higher mortality rate associated with NTFx among adults with vs. without CP. In summary, the risk factors for acute and long-term post-NTFx mortality risk among adults with CP may encompass general risk factors, such as pre- or post-NTFx chronic diseases, but there may also be factors unique to the medical complexity of CP, such as the type and severity of CP, use of assistive walking device or wheelchair, and medication use and polypharmacy. Future studies are needed to disentangle the factors that may differentially associate with post-NTFx mortality, and to what extent the NTFx-mortality association is mediated by modifiable (e.g., physical activity) or treatable (e.g., pneumonia, metabolic disease) complications.

The limitations of this study must be discussed. We were unable to account for the severity of CP and type of CP as this information is not available in claims, and unable to obtain information regarding socioeconomic status (e.g., income, education). However, severity of CP is associated with an elevated burden of disease. Therefore, adjusting for an array of chronic diseases limits the potential confounding, to some extent, by lack of severity information of CP and some socioeconomic status indicators. Further, based on comparisons between clinical- and claims-based studies for adults with CP, the private insurance sample of adults with CP reflects a disease prevalence similar to those with mild to moderate forms of CP, whereas the public insurance sample of adults with CP reflects a disease prevalence similar to those with moderate to severe forms of CP (Whitney et al., 2018b; Whitney, 2019c; Whitney et al., 2019g; French et al., 2019b). Despite the differences in health and disease status, risk of fracture does not differ between privately or publicly insured (Medicare) adults with CP (Whitney et al., 2020). This may be explained on a functional level. While the skeletal system is more fragile for more severe forms of CP, activities, events, and lifestyles differ dramatically for adults with CP based on functional ability, which may impact the likelihood of a fracture event independent of the degree of skeletal fragility. Further evidence to support a lack of an association between severity of CP and fracture risk among adults comes from Trinh et al. (Trinh et al., 2016) who reported no association between ambulatory status and adult-onset fracture; although, the number of fractures (*n* = 17) and total sample (*n* = 45) in their study was small. Taken together, the lack of CP severity information likely has a negligible impact on the broader conclusions drawn from this study. Nevertheless, future studies are needed to examine patterns by severity of CP and socioeconomic status indicators. Further, we were unable to statistically adjust for race due to the extent of missingness/unknown from Optum and differences in coding between Optum and Medicare. Future studies are needed that fully examine disparities by race on the contribution of skeletal fragility to unhealthful aging for adults with CP. This study included adults without CP as a comparison from the Optum database, which may introduce some bias due to the differences in enrollment criteria, such as affluent and employment status. However, we aimed to provide a non-CP comparison group that was representative of the adult general population without disabilities that would lead to Medicare entitlement. While the comorbidities examined in this study have shown good accuracy for identification, it is unknown if there are substantive differences in the diagnostic accuracy of these conditions between adults with and without CP. While we were able to ascertain a large, nationwide sample of adults with CP from private (Optum) and public (Medicare) administrative claims databases, we did not have access to Medicaid claims, which typically provides coverage for “sicker” individuals with greater medical needs. Given the differences in insurance enrollment criteria and the medical needs of adults with CP, not having Medicaid claims data may have resulted in conservative estimates, and the public health issue may be larger than what this study can capture.

## 5 Conclusion

Study findings suggest that NTFx is a risk factor for mortality among adults with CP, and compared to adults without CP, NTFx is associated with a greater mortality rate for up to 12 months for adults with CP. Further, while NTFx of the vertebral column and hip elicit the highest mortality rates compared to extremity sites, NTFx of the vertebral column and lower extremities may elicit a higher relative rate of mortality for adults with vs. without CP. Future clinical research is needed to identify strategies to prevent and better manage skeletal fragility with the aim of reducing the burden of skeletal fragility and improving healthful aging for adults with CP. Future basic and translational studies are needed that examine mechanisms linking skeletal fragility with mortality specific to the population of adults with CP.

## Transparency document

Transparency document. Image 1

## CRediT authorship contribution statement

Daniel G. Whitney: Conceptualization, Methodology, Validation, Formal analysis, Investigation, Resources, Writing- Original Draft, Writing- Review & Editing, Visualization, Supervision, Project Administration, Funding acquisition. Sarah Bell: Methodology, Software, Validation, Investigation, Data Curation. Edward A. Hurvitz: Writing- Review & Editing, Data and Clinical Interpretation. Mark D. Peterson: Writing- Review & Editing, Data and Clinical Interpretation. Michelle S. Caird: Writing- Review & Editing, Data and Clinical Interpretation. Karl J. Jepsen: Writing- Review & Editing, Data and Clinical Interpretation.

## Declaration of competing interest

None.
